# Commentary: The Role of Dentists and Primary Care Physicians in the Care of Patients With Sleep-Related Breathing Disorders

**DOI:** 10.3389/fpubh.2018.00354

**Published:** 2018-12-04

**Authors:** Omar F. Suarez

**Affiliations:** Department of Dental Medicine, Postdoctoral Residency Program, Hansjörg Wyss Department of Plastic Surgery, NYU School of Medicine, Brooklyn, NY, United States

**Keywords:** rheumatology, autoimmune disorders, systemic inflammatory diseases, orofacial care, dental screening

I read with interest the article on the need for collaboration between dentists and primary care physicians in the care of patients with sleep-related breathing disorders (1). The objective of this General Commentary is to underscore the importance of interprofessional education and practice and emphasize the benefits, exemplified by orofacial pain disorders presenting secondarily to systemic inflammatory conditions.

Clinical rheumatologists are challenged by the complexity of autoimmune disorders and systemic inflammatory diseases. Scientific advances over the last 20 years have increased understanding of the pathophysiology of these conditions and improved strategies to relieve pain and discomfort. As the numbers of patients screened, diagnosed, and treated has increased, so too has the need for interprofessional education and practice to improve the lives of patients. Arthritis is the leading cause of disability, (2) presently affecting ~25% of the US population with projected estimates of 54 million affected people by the year 2040 (3).

It is important to clarify what is meant by the term *arthritis*. A general yet limited understanding is that arthritis denotes painful conditions affecting joints. Nonetheless, rheumatology uses the term arthritis more broadly to indicate systemic inflammatory conditions that are not restricted to joints *per se* (4).

Rheumatic conditions are broadly categorized into those with known positive serological biomarkers (i.e., seropositive conditions) and those with no known serological biomarkers (i.e., seronegative conditions). Regardless of their classification, rheumatic conditions are progressively debilitating. More favorable outcomes are predicated on early recognition and evidence-based intervention.

Perhaps not surprisingly, seropositive disorders lend themselves to earlier and more definitive diagnosis. Seronegative conditions, however, are more elusive; diagnosis often relies on clinical presentation patterns or tissue changes. Unfortunately, at later stages of detection, the tissue changes are often irreversible, prompting a global effort toward earlier recognition of these disorders to minimize pain and disability for patients. A subset of these conditions affect ligaments, tendons, joint capsules, and insertional attachments of muscles to bones and bursae (4).

The structures constituting the connective tissue between tendon or ligament and bone are collectively referred to as enthesis (5). In recent years, enthesitis (i.e., inflammation of the enthesis) has garnered attention as a potentially important clinical flag for the presence of systemic inflammatory conditions (6). Several enthesitis indices are currently in use to quantify patterns of entheseal involvement (7). Strikingly absent from all these indices are structures (i.e., entheses) of the orofacial region.

While there is a body of literature on the orofacial consequences of certain rheumatologically related conditions, including rheumatoid arthritis and Sjögren's syndrome, (8–10) there is a void in the scientific corpus regarding recognition of orofacial and dental disorders related to seronegative rheumatic disorders. Most interventions offered by dentistry attempt to restore function that has been lost, rather than prevent the untoward effects of progressive disease processes.

Orofacial structures that meet the anatomical and physiological criteria of entheses are not currently listed in screening and assessment tools for rheumatic conditions. These potentially include the stylomandibular and sphenomandibular ligaments, the temporomandibular (TM) joint proper, and joint capsules, e.g., the aponeurosis of the masseter and temporalis muscles, the temporalis tendons, the insertion of the sternocleidomastoid (SCM), and the periodontal ligament.

Patients with orofacial or dental entheseal inflammation present a challenge to the mechanically favored diagnostic approaches in dentistry. Such patterns of inflammation are generally difficult to diagnose and rarely respond to dental intervention on a timely basis. As a result, patients may undergo additional dental treatment that is both misguided and ineffective. As the protocols in pain management are being revisited in the face of a national opioid epidemic, improved understanding of the underlying mechanisms of pain are paramount to delivering effective care to patients. Discerning the perception of pain alone is an insufficient and unacceptable professional standard.

Dentists know first-hand about the complexity of orofacial structures. We also need to appreciate the pathophysiology of inflammatory disorders and how they affect tissues and organs. As professionals devoted to prevention, through interprofessional collaborations and interdisciplinary research, we will be in stronger positions to investigate how rheumatic conditions concurrently manifest themselves in the orofacial region.

Three overlapping priorities in need of concerted interprofessional attention toward using orofacial care to improve the lives of patients with rheumatic conditions are proposed next. First, studies are needed to determine the prevalence of orofacial (dental) disorders in patients with known inflammatory conditions. Second, screening protocols at chairside ought to be developed and clinically and scientifically tested to detect orofacial (dental) signs and symptoms that may ultimately lead to the diagnosis of systemic inflammatory conditions. Third, patients with known auto-inflammatory conditions who are being managed with medications that modulate immune system responses ought to be monitored closely by dentists for opportunistic infections and cancers of the mouth and supporting structures.

The following recommendations are offered to better ensure that the primary care and dental communities are part of the holistic care of patients with rheumatic conditions.

1. Create a repository of medical / dental observations in patients with systemic inflammatory diseases, perhaps via a competitive grant awarded through the US National Institutes of Health. This would include, at a minimum, a protected online portal for contributions by providers and access to shared information to interested research and therapeutic communities.
2. Establish protocols of orofacial (dental) assessments of patients with known systemic inflammatory conditions.
3. Ensure access to tailored interprofessional practice / interprofessional education (IPP/PE) learning modules for medical and dental professionals.
4. Support the formation of specialty dental / orofacial clinics associated with rheumatology departments.
5. Develop continuing dental education (CDE) modules for rheumatic conditions and their effects on orofacial structures.

To begin, it may be important for the particular dental (e.g., orofacial pain dentists) and medical professionals (e.g., rheumatologists) with a front row seat to the challenges patients experience (e.g., pain and debilitation from systemic inflammatory disease) to collaborate. Eventually, increased awareness will lead to interprofessional education and in turn to upgrades in interprofessional practice, broadening the scope of those involved in providing care and increasing the benefits to the patients and their families.

## Author Contributions

The author confirms being the sole contributor of this work and has approved it for publication.

### Conflict of Interest Statement

The author declares that the research was conducted in the absence of any commercial or financial relationships that could be construed as a potential conflict of interest.
